# Cataract Surgery in Dry Eye Disease: Visual Outcomes and Complications

**DOI:** 10.3389/fmed.2020.575834

**Published:** 2020-10-07

**Authors:** Pragnya R. Donthineni, Anthony V. Das, Swapna S. Shanbhag, Sayan Basu

**Affiliations:** ^1^The Cornea Institute, L V Prasad Eye Institute, Hyderabad, India; ^2^Department of eyeSmart EMR & AEye, L V Prasad Eye Institute, Hyderabad, India; ^3^Center for Ocular Regeneration (CORE), L V Prasad Eye Institute, Hyderabad, India

**Keywords:** dry eye disease (DED), cataract surgery, Stevens–Johnson syndrome (SJS), Sjogren's syndrome, electronic medical records (EMR)

## Abstract

**Purpose:** To describe the visual outcomes and complications following cataract surgery in dry eye disease (DED).

**Methods:** This retrospective study included 668 eyes of 399 patients with DED, who underwent cataract surgery between 2011 and 2019 at our multi-tier ophthalmology hospital network. Based on etiology, they were divided into three groups: cicatrizing conjunctivitis (CC), meibomian gland dysfunction (MGD), and Sjogren's syndrome (SS). The data on demographics, visual impairment, surgical technique, visual outcomes, and complications were collected using an electronic medical record system. Median LogMAR best corrected visual acuity (BCVA) with interquartile range (IQR) was compared using Wilcoxon's rank sum test.

**Results:** The median age at which cataract surgery was performed was 58 (IQR: 47–65) years. Etiology of DED was CC in 279, MGD in 255, and SS in 134 eyes. Most (471) eyes underwent phacoemulsification, under peribulbar anesthesia (548) through a temporal clear corneal incision (209) with foldable intraocular lens implantation (417). The overall median LogMAR BCVA improved from 1.1 (IQR: 0.6–2.1) at baseline to 0.3 (IQR: 0.1–0.7) and 0.1 (IQR: 0–0.65) at 1 and 6 weeks (*p* < 0.0001) post-operatively. The median 6 weeks post-operative BCVA was 0.3, 0.1, and 0.1 in CC, MGD, and SS, respectively, and significantly better than at baseline (*p* < 0.0001). The leading cause of sub-optimal vision was corneal scarring (44; 9%), and the most common complication was posterior capsular rupture with vitreous loss (23; 3%).

**Conclusion:** Cataract surgery has good visual outcomes in patients with DED, without any disconcerting rate of complications. Pre-existing keratopathy is the main determinant of the extent of post-operative visual recovery.

## Introduction

Dry eye disease (DED) comprises a spectrum of disorders which cause tear film instability, hyperosmolarity, chronic inflammation, and neurosensory abnormalities, all of which lead to chronic ocular surface dysfunction (1). In addition to causing keratopathy, preexisting DED poses challenges during cataract surgery, especially in the presence of chronic inflammation, cicatrizing changes, lid abnormalities, and compromised media clarity (2). It is also known to worsen following cataract surgery leading to a higher rate of complications and compromised visual outcomes (3–6). This is probably due to increased inflammation, toxicity from eye drops containing preservatives like benzalkonium chloride (BAK), and corneal nerve damage from limbal incisions. Besides, phacoemulsification, by itself, has been reported to cause a reduction in tear film breakup time, tear meniscus height, and corneal sensitivity post-operatively (7).

Studies done in larger cohorts in India have reported an increased incidence of DED with age, which is also the pattern seen with cataract (8, 9). It is hence not uncommon to encounter situations where we need to either simultaneously or sequentially manage both dry eye and cataract. In order to achieve successful results, the need to understand the various factors governing the outcomes of cataract surgery in eyes with pre-existing DED cannot be overemphasized. Most studies in the existing literature have looked at outcomes of cataract surgery in specific disorders causing severe DED in smaller cohorts (10–12). Thus, in the current study, the authors have attempted to study the etiology-based visual outcomes and complications of cataract surgery in a larger cohort of patients with DED.

## Materials and Methods

### Study Design, Period, Location, and Approval

This was a retrospective study that included 668 eyes of 399 patients diagnosed with dry eye disease who underwent cataract surgery between August 2010 and May 2019 at a multi-tier ophthalmology hospital network in India (13). The study was approved by the Institutional Ethics Committee and adhered to the Declaration of Helsinki. A standard consent form including consent for electronic data privacy was obtained from all the patients. The clinical data of each patient who underwent a comprehensive ophthalmic examination was entered into a browser-based electronic medical records system (eyeSmart EMR) by uniformly trained ophthalmic personnel and supervised by an ophthalmologist using a standardized template (14).

### Cases, Data Retrieval, and Classification

The eyeSmart EMR was screened for patients with symptoms, signs, referral, or final diagnosis or management suggestive of DED. The detailed process of identifying patients with a diagnosis of DED has been described (8). Among these patients, only those with both symptoms and signs of DED were included in the study as per the recommendations of the tear film and ocular surface dry eye workshop (TFOS DEWS) II (15). Complaints of dryness, grittiness, irritation, foreign body sensation, or discomfort, not associated with severe pain, sudden vision loss, itching, discharge or signs of allergy, contact lens wear, or recent systemic infection were considered positive for DED symptoms. Those with fluorescein breakup time (FBUT) <10 s, corneal fluorescein staining of >5 spots, Schirmer's test *I* ≤ 10 mm/5 min, abnormal meibomian gland function (poor expressibility, blockage, or atrophy) were considered as positive for signs of DED.

Among these patients with preexisting DED, those who underwent cataract surgery during the study period were included in this study. These patients were classified into evaporative dry eye disease (MGD) and aqueous deficiency dry eye (ADDE) based on TFOS DEWS II classification (15). All the patients with ADDE underwent an evaluation by the internist to diagnose the underlying systemic association. Based on etiology, they were further divided into three groups as (i) meibomian gland dysfunction (MGD), (ii) Sjogren's syndrome (SS), and (iii) cicatrizing conjunctivitis (CC). Those with systemic examination and serology suggestive of Sjogren's syndrome (primary or secondary) were included in group SS. All patients of ADDE with signs of conjunctival cicatrization were diagnosed and included into CC group based on an algorithmic approach including a detailed history, clinical examination, and diagnostic workup that has been described (16, 17). The data on age, gender, best corrected visual acuity (BCVA) at presentation, type of cataract surgery performed, complications, and post-operative BCVA at 1 and 6 weeks were documented and extracted using the eyeSmart EMR. The main outcome measures considered were post-operative distance BCVA as per WHO guidelines (18) and the rate and type of complication post-cataract surgery.

### Interventions and Post-operative Follow-Up

**Figure 1:**
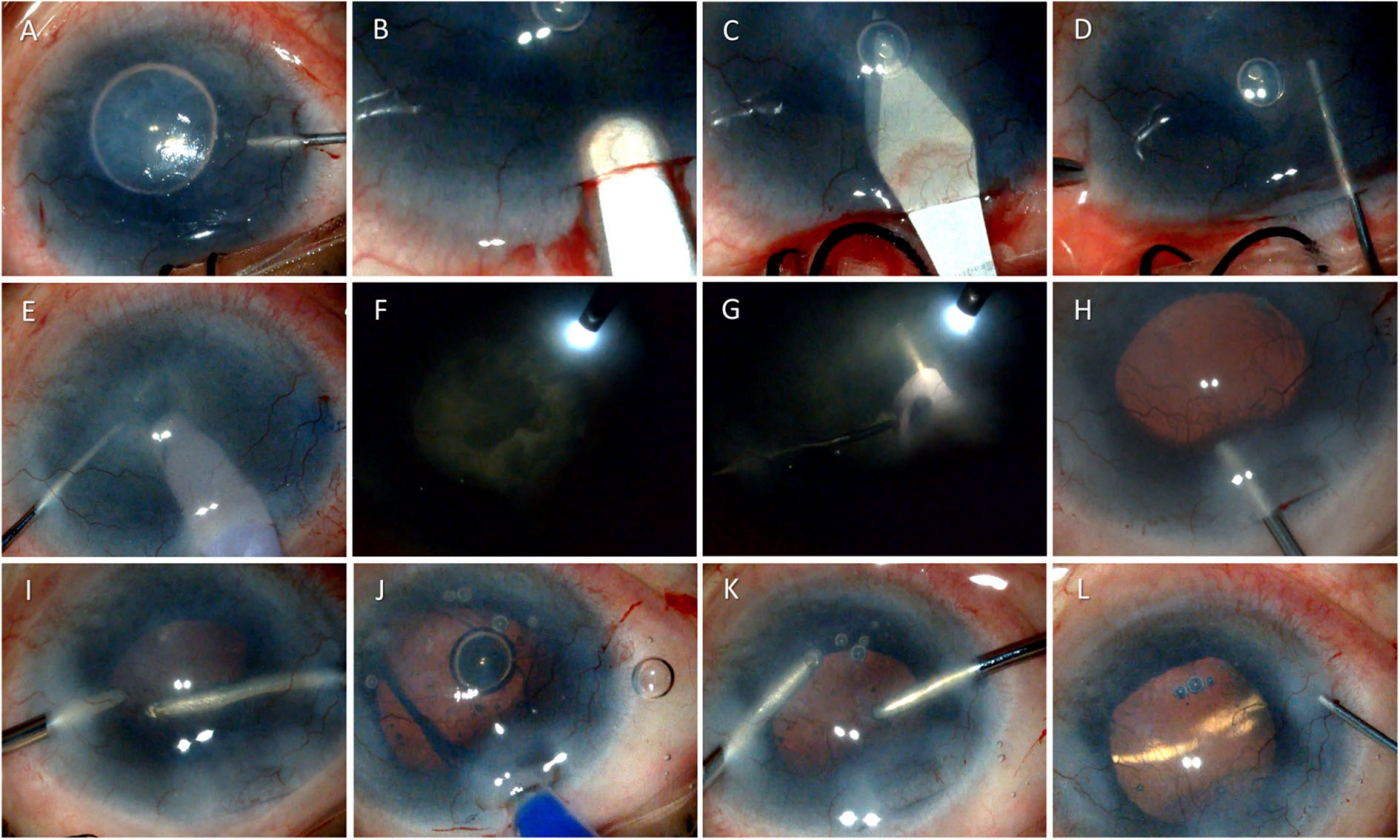
Surgical technique of phacoemulsification in a case of dry eye disease due to ocular cicatrizing pemphigoid. The figure shows serial photomicrographs demonstrating the steps of cataract surgery in a 54-year-old patient with ocular cicatrizing pemphigoid (OCP). Image **(A)** shows injection of Trypan blue through a paracentesis at 10 o'clock position during cataract surgery to enhance visualization of the anterior capsule, followed by a 2.8 mm superior clear corneal incision using a crescent blade **(B)** and anterior chamber entry using a 2.8 mm keratome knife **(C)**. Continuous curvilinear capsulorhexis is being performed using a cystitome **(D)** followed by nucleus management using stop and chop technique **(E)** under endoilluminator assistance **(F)** and **(G)**. Image **(H)** shows injection of viscoelastic into the anterior chamber and clear visualization of the posterior capsule under retro illumination. The bottom row shows cortical aspiration using a bimanual irrigation aspiration cannula **(I)**, foldable intraocular lens implantation **(J)**, and removal of viscoelastic from the anterior chamber **(K)** prior to wound hydration and closure **(L)**.

All the patients were taken up for cataract surgery after optimization of the ocular surface. The standard criteria considered to decide on the type of anesthesia were (i) age of the patient, (ii) patient cooperation, (iii) pupillary dilatation, (iv) corneal clarity, (v) ability to tolerate microscopic light (vi), grade of cataract, and (vii) presence of symblepharon. In cooperative patients with good media clarity and pupillary dilatation, topical anesthesia was administered. Peribulbar blocks were administered in patients with compromised media clarity, poor pupillary dilatation, inability to cooperate, hard cataracts, and when long surgical time was anticipated. A mixture of 4–5 mL lidocaine hydrochloride (2%) and 2–3 mL bupivacaine hydrochloride (0.5%) were used for peribulbar injection. Intraocular lens (IOL) power calculation was done using laser interferometric biometry (LENSTAR). Keratometry (K) readings from corneal topography were used in case of steep corneas, and standard K readings were used when they could not be captured. The cases with advanced keratopathy, who were already scleral contact lens users with unreliable keratometry and IOL power calculation, were left aphakic, and the residual refractive error was subsequently incorporated into the scleral contact lens. Prior to surgery, povidone iodine (5%) was used to disinfect conjunctiva and the skin around the eye. Phacoemulsification was performed in eyes with adequate media clarity, good pupillary dilatation, and even in eyes with moderate to severe keratopathy with the assistance of an endoilluminator (Figure 1). In eyes with significant corneal scarring, hard cataracts, significant symblepharon, a manual small incision cataract surgery (MSICS) with Blumenthal technique was performed. Extracapsular cataract extraction (ECCE) was performed in eyes with very poor media clarity. Scleral incisions were taken in eyes with peripheral corneal thinning and temporal approach and lateral canthotomy, or lid sutures were taken in cases with poor exposure. Special intraoperative precautions for dry eyes like (i) liberal application of dispersive viscoelastic in the form of 2% hydroxy-propyl methylcellulose (HPMC) on the ocular surface, after speculum application and throughout surgery, (ii) incisions avoiding areas of corneal scarring and thinning, (iii) coaxial illumination/red reflex enhancer, (iv) use of trypan blue (0.06%) for capsulorhexis, and (v) therapeutic bandage contact lens (BCL) at the end of the surgery (which was removed at 1 week) were undertaken based on the severity of keratopathy due to DED. Wound closure was done in all cases with irregular or thin corneas with interrupted 10-0 monofilament nylon sutures. Post-operative regimen included prednisolone acetate (1%) eye drops six times a day, tapered by one drop each week for 6 weeks, and moxifloxacin hydrochloride (0.5%) four times a day for 1 week. Other medications like topical lubricants, cyclosporine, and systemic medications that were being used pre-operatively were continued. Patients were seen on post-operative day 1, week 1, and 6 weeks.

### Statistical Analysis

Descriptive statistics using mean (± standard deviation) and median with inter-quartile range (IQR) were used to elucidate the demographic data. All tables for age, gender, etiological diagnosis, and outcomes were drawn and analyzed using Microsoft Excel (Microsoft Corporation, Redmond, USA). Median distance LogMAR BCVA with IQR was calculated for each group and post-operative visit and compared using Wilcoxon's signed-rank test for paired samples.

## Results

### Demographics and Etiology of DED

The median age of DED patients undergoing cataract surgery was 58 years (IQR: 47–65). Among the 399 cases, 265 (66.42%) and 134 (33.58%) were females and males, respectively. Of the 668 eyes included in the study, CC was the most common cause of DED seen in 279 (41.77%) eyes followed by MGD and SS, which were seen in 255 (38.17%) and 134 (20.06%) eyes, respectively. Of the 279 eyes with CC, Stevens–Johnson syndrome (SJS) was the leading cause seen in 209 (31.29%) eyes followed by ocular cicatricial pemphigoid (OCP) and graft-versus-host disease (GVHD) in 59 (8.8%) and 1 (0.15%) eye. The cause of cicatrization was unknown in 10 (1.5%) eyes. Among the 134 (20.06%) eyes with SS, primary and secondary SS was diagnosed in 19 (2.84%) and 115 (17.22%) eyes, respectively.

### Technique and Details of Cataract Surgery

**Figure 2:**
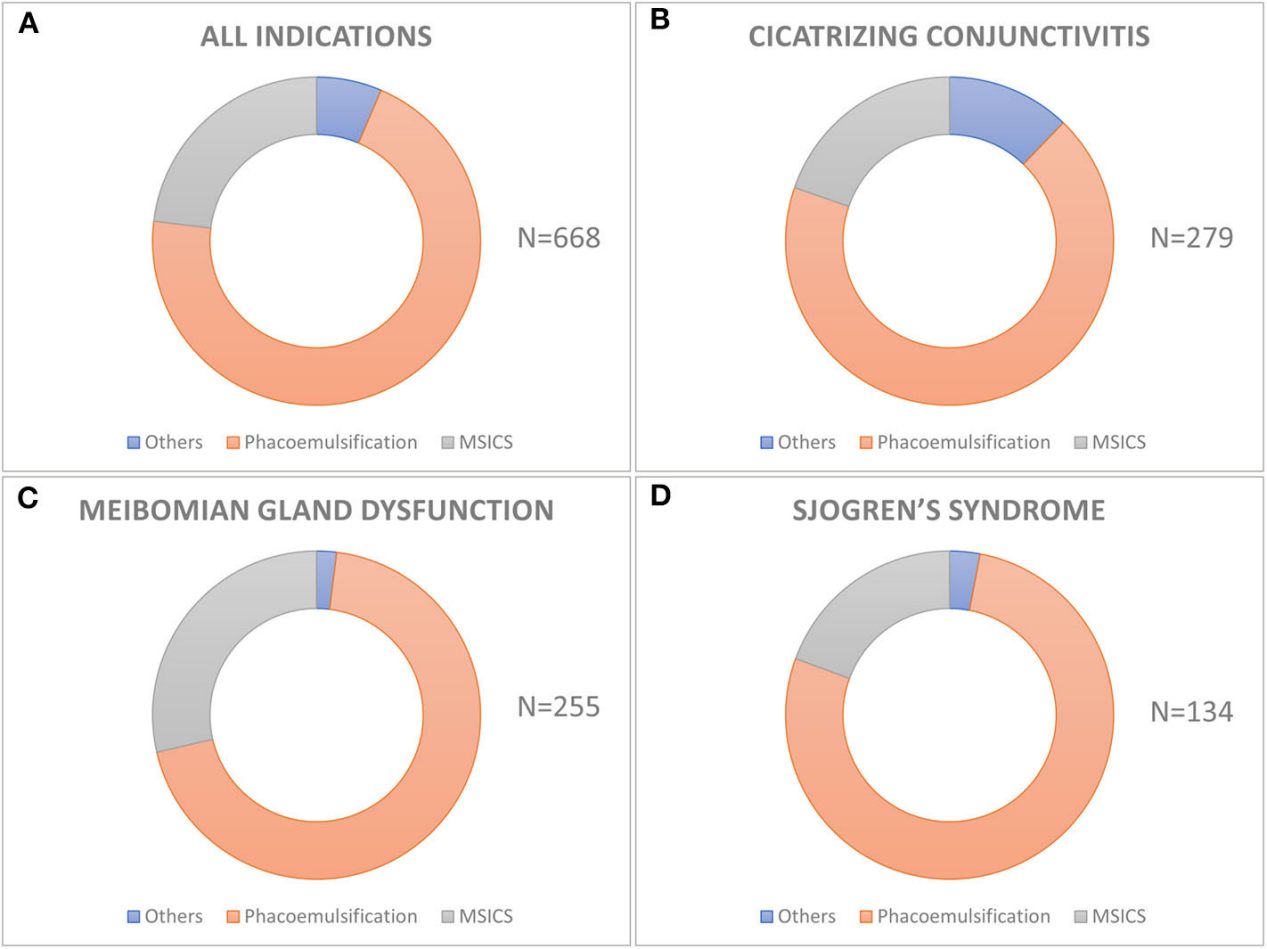
Techniques of cataract surgery used in different etiologies of dry eye disease. The donut graph shows the distribution of various techniques of cataract surgery in the entire cohort **(A)** and in different etiologies of dry eye like cicatrizing conjunctivitis **(B)**, meibomian gland disease **(C)**, and Sjogren's syndrome **(D)**. Phacoemulsification is the most common technique of cataract surgery performed in all the three groups and also in the entire cohort.

Of the 668 eyes, phacoemulsification was the most common surgical procedure performed in 471 (70.5%) eyes followed by manual small incision cataract surgery (MSICS) in 154 (23.05%), extracapsular cataract extraction (ECCE) in 42 (6.3%), and intracapsular cataract extraction (ICCE) in 1 (0.15%) eye (Figure 2). Peribulbar anesthesia was used in 548 (82.04%), topical in 106 (15.87%), and general anesthesia was required in 14 (2.1%) cases. Of the 471 eyes which underwent phacoemulsification, it was performed through a 2.8 mm clear corneal incision in 304 (64.5%; 209/68.75% of which were temporal), limbal incision in 35 (7.4%), and scleral incision in 132 (28.1%) eyes, while all MSICS and ECCE surgeries were done through (5–8 mm) superior scleral incisions. A continuous curvilinear capsulotomy was used in 512 (76.65%, 6/1.2% of which were femtosecond laser-assisted), can-opener in 151 (22.6%), and envelope in 5 (0.75%) eyes. The nucleotomy technique in 471 eyes undergoing phacoemulsification was stop and chop in 257 (54.6%), direct vertical chop in 121 (25.7%), divide and conquer in 36 (7.64%), phaco-aspiration in 51 (10.8%), chip and flip in 3 (0.63%), and phaco plow in 3 (0.63%). All MSICS surgeries were performed using the Blumenthal technique. The irrigating solution used was ringer lactate in 493 (73.8%) and balanced salt solution in 175 (26.2%) eyes. The intra-ocular visco-surgical devices used were 2% hydroxy-propyl methylcellulose (HPMC) in 552 (82.63%) eyes and hyaluronic acid 10 mg/mL in the remaining 116 (17.37%) eyes. There were 44 (6.5%) eyes that were left aphakic, while 417 (62.43%) eyes were implanted with a foldable acrylic IOL, and 207 (30.99%) eyes had a rigid Polymethyl methacrylate (PMMA) IOL implanted. Among the 417 eyes with foldable acrylic IOLs implanted, 374 were non-aspheric monofocal (89.7%), 25 (5.99%) were aspheric monofocal, 14 (3.36%) were aspheric multifocal, 2 (0.48%) were aspheric toric, and 2 (0.48%) were aspheric toric-multifocals.

### Visual Outcomes

**Figure 3:**
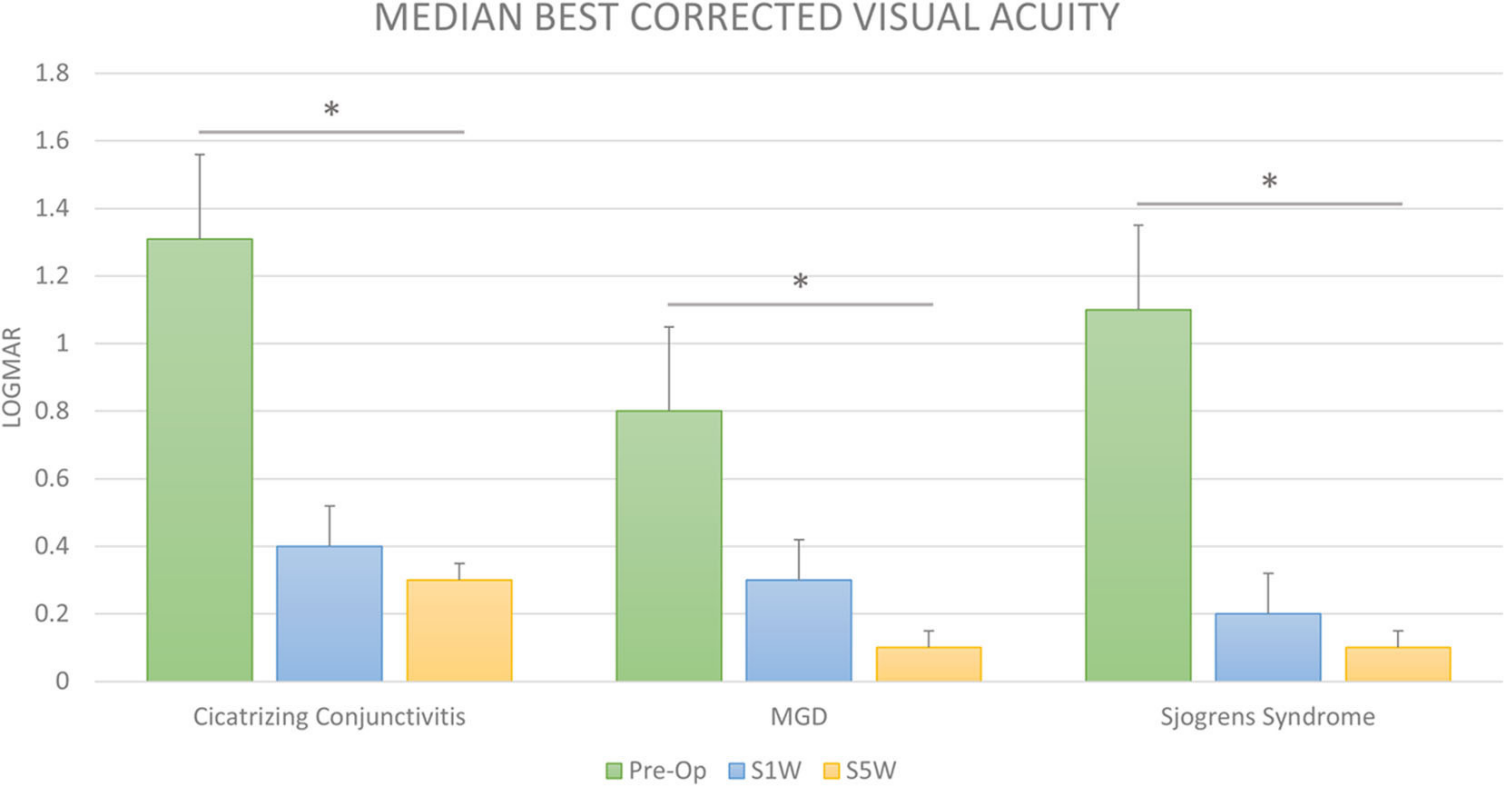
Visual outcomes after cataract surgery in dry eye disease. The bar diagram shows the median LogMAR corrected distance visual acuity (BCVA) prior to cataract surgery (Pre-op) and at 1 (S1W) and 6 weeks (S6W) post-surgery in cicatrizing conjunctivitis (CC), meibomian gland disease (MGD), and Sjogren's syndrome (SS). The visual improvement was significant and gradually increased up to 6 weeks post-surgery from baseline in all the three groups. *Statistically significant difference (Wilcoxon's signed-rank test).

The overall median pre-operative LogMAR BCVA was 1.1 (IQR: 0.6–2.1). The BCVA improved to a median of 0.3 (IQR: 0.1–0.7) at post-operative 1 week (Figure 3). Further improvement was seen at the final post-operative visit at 6 weeks (Figure 3), with a median of 0.1 (IQR: 0–0.65). To put this in Snellen equivalents, the overall median BCVA improved from 20/250 pre-operatively to 20/25 at 6 weeks post-operatively (*p* < 0.0001).

### Complications

**Figure 4:**
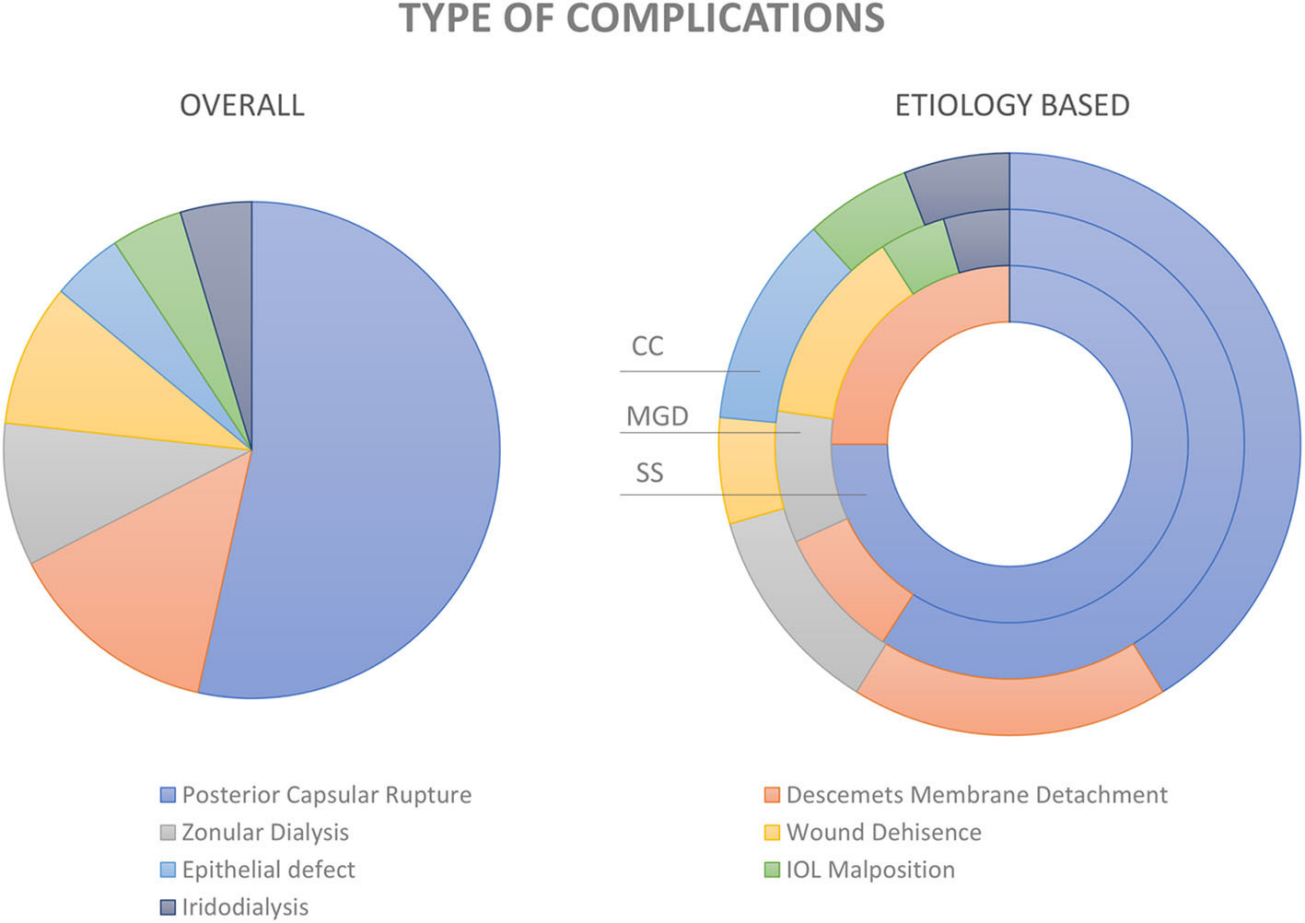
Complications of cataract surgery in dry eye disease. Pie diagram showing the distribution of various types of complications in the entire study cohort (image on the left). The distribution within the groups; i.e., cicatrizing conjunctivitis (CC), meibomian gland dysfunction (MGD), and Sjogren's syndrome (SS) are depicted in the donut graph (image to the right).

Surgical complications occurred in 43 (6.44%) of the 668 eyes (Figure 4). Intra-operative complications included posterior capsular rupture (PCR) with vitreous loss in 23 (3.44%), zonular dehiscence (ZD) in 4 (0.6%), and iridodialysis in 2 (0.3%) eyes. Post-operative complications included Descemet's membrane detachment (DMD) in 6 (0.9%), wound dehiscence/leakage in 4 (0.6%), corneal epithelial defect in 2 (0.3%), and intraocular lens (IOL) malposition in 2 (0.3%) eyes. There were no cases of immediate or late onset surgical site infection or endophthalmitis noted in this cohort.

### Causes of Sub-optimal Visual Recovery

Of the 668 eyes that underwent cataract surgery, low vision (≤20/80) was seen in 61 (9.13%) eyes, all of which were related to keratopathy. The most common cause was preexisting corneal scarring in 44 (72.13%) eyes followed by corneal decompensation, epithelial irregularities, keratinization of the corneal surface, and corneal vascularization seen in 5 (8.2%), 4 (6.56%), 2 (3.28%), and 1 (1.63%) eye, respectively. The cause for low vision was not discernable in 5 (8.2%) eyes.

### Cicatrizing Conjunctivitis (*n* = 279)

Phacoemulsification was the most common surgical procedure, performed in 190 (68.1%) eyes, followed by MSICS in 55 (19.71%), ECCE in 33 (11.83%), and ICCE in 1 (0.36%) case. The median pre-operative LogMAR BCVA was 1.31 (IQR: 0.8–2.8). The median LogMAR BCVA at post-operative 1 week was 0.4 (IQR: 0.1–1) and at 6 weeks post-operative was 0.3 (IQR: 0–1). To put this in Snellen equivalents, the overall median BCVA improved from 20/400 pre-operatively to 20/40 at 6 weeks post-operatively (*p* < 0.0001). Surgical complications were encountered in 22 (7.89%) eyes. PCR with vitreous loss was the most common complication seen in 13 (4.65%) eyes followed by wound dehiscence, DMD, ZD, IOL malposition, and iridodialysis seen in 3 (1.08%), 2 (0.72%), 2 (0.72%), 1(0.36%), and 1(0.36) eyes, respectively.

### Meibomian Gland Dysfunction (*n* = 255)

Phacoemulsification was the most common surgical procedure performed in 177 (69.4%) eyes followed by MSICS in 73 (28.63%) and ECCE in 5 (1.96%) cases. The median pre-operative LogMAR BCVA was 0.8 (IQR: 0.5–1.5). The corresponding median LogMAR BCVA at post-operative 1 week was 0.3 (IQR: 0.1–0.6), and that at surgical 6 weeks was 0.1 (IQR: 0–0.3). To put this in Snellen equivalents, the overall median BCVA improved from 20/125 pre-operatively to 20/25 at 6 weeks post-operatively (*p* < 0.0001). The surgical complications were encountered in 17 (6.67%) eyes. PCR with vitreous loss was the most common complication seen in 7 (2.74%) eyes followed by DMD, corneal epithelial defect, wound dehiscence, ZD, IOL malposition, and iridodialysis seen in 3 (1.18%), 2 (0.78%), 2 (0.78%), 1 (0.36%), 1 (0.36%), and 1 (0.36) eyes, respectively.

### Sjogren's Syndrome (*n* = 134)

Phacoemulsification was the most common surgical procedure performed in 104 (77.61%) eyes followed by MSICS in 26 (19.4%) and ECCE in 4 (2.99%) cases. The median pre-operative LogMAR BCVA was 1.1 (IQR: 0.65–1.9). The median LogMAR BCVA at post-operative 1 week was 0.2 (IQR: 0–0.5) and at post-operative 6-weeks was 0.1 (IQR: 0–0.4). To put this in Snellen equivalents, the overall median BCVA improved from 20/250 pre-operatively to 20/25 at 6 weeks post-operatively (*p* < 0.0001). The surgical complications were encountered in 4 (2.98%) eyes. PCR with vitreous loss was the most common complication seen in 3 (2.24%) eyes followed by DMD seen in 1 (0.75%) eye.

## Discussion

In the presence of DED, where there is loss of homeostasis with a hostile environment, an otherwise routinely performed intervention like cataract surgery becomes quite challenging (19). Thus, in the current study the authors aimed to look at the etiology-based visual outcomes and complications following cataract surgery in eyes with preexisting DED. This study found that overall, the visual outcomes of cataract surgery, irrespective of the etiology of DED, were good and without any alarming rate of post-operative complications. All cases of sub-optimal visual recovery had some degree of keratopathy, particularly corneal scarring, although the degree of keratopathy could not be quantified and correlated with the visual outcomes.

The mean age at which patients with DED underwent cataract surgery was found to be 55 years, which was much early as compared to 70–75 years reported for most routine cataract surgeries (20–22). This is not surprising and is because of associated chronic inflammation, long-term and/or frequent use of topical or even systemic steroids, early presentation due to keratopathy, or early surgical planning for ease of surgery (23). Early surgical intervention after stabilizing the ocular surface is preferred especially in patients with SJS and OCP; as both the density of cataract and keratopathy progress with time making surgical intervention more onerous (11, 23). Phacoemulsification was the most common type of surgery performed in all the three categories of DED and is a feasible option when planned before media clarity gets significantly compromised (10, 23, 24). While phacoemulsification can still be performed in eyes with significant keratopathy with the assistance of an endoilluminator (10, 25), ECCE needs to be performed in the presence of severe keratopathy (23). Two thirds of the eyes which needed ECCE in our cohort had dry eye associated with CC, which implies a higher level of surgical complexity. Posterior capsular rupture with vitreous loss was the most common complication noted in all the three groups, which is expected in a setting of compromised media clarity. Thus, judicious use of viscoelastic and trypan blue to enhance visualization of the anterior capsule may help minimize this complication (26). DMD was noted in 0.9% of the operated eyes, which was limited to the incision sites and resolved without any surgical interventions. Though noted in a small proportion of patients, it should be watched for cautiously intraoperatively, to avoid the need for surgical intervention in these eyes which are at high risk for failure following corneal transplant (27). Though corneal melt following cataract surgery has been reported in some studies, especially with use of non-steroidal anti-inflammatory drug (NSAIDs) eye drops, we did not encounter this complication in any of the eyes that were treated with them (3, 28, 29).

Another interesting finding noted in this study was the pattern of visual recovery at first and sixth weeks following cataract surgery. While the BCVA improved gradually up to 6 weeks post-surgery in MGD and SS; in eyes with CC, the visual recovery was similar at 1 and 6 weeks post-surgery. This was consistent with the characteristic pattern of visual recovery reported previously in patients with SJS (23). Most findings in this study like mean age at surgery, choice of surgical technique, and complications corroborated with that reported in smaller cohorts of either SJS/OCP or SS, and an overview of literature on visual outcomes and complications following cataract surgery in DED is described in Table 1.

**Table 1:** Overview of literature on visual outcomes and complications following cataract surgery in dry eye disease (DED).

**References**	**No of eyes (*n*)**	**Diagnosis**	**Mean age** **(years)**	**Type of** **surgery**	**Pre-op mean** **LogMAR BCVA**	**Post-op mean** **LogMAR BCVA**	**Complications (*n*; %)**
Narang et al. (23)	40	SJS	–	Phaco ECCE	1.61	1 month–0.30 LFU–0.48	PCR (2/40; 5%) Ocular surface breakdown (4/40; 10%)
Sharma et al. (10)	21	SJS	32 ± 12	Phaco	2.29	1 month: 0.84	CED (2/21; 9.5%) and filamentary keratitis (2/21; 9.5%)
de Melo et al. (24)	72	GVHD	56	Phaco	0.39	1 year: 0.08	CME (4/72; 5.5), keratitis with perforation (2/72; 2.7%)
Puranik et al. (30)	9	OCP	60 ± 2	ECCE Phaco	–	>2 lines improvement in 6 of 9 eyes at 1 year	SPK (1/9; 11%), CED (1/9; 11%), CME (1/9; 11%)
Ram et al. (6)	25	Non-specific	59 ± 10	Phaco	–	0 (13 eyes) 0.2–0.5 (8 eyes) 0.5–1 (4 eyes)	SPK (8/25; 32%) and CED (8/25; 32%)
Geerling et al. (31)	15	OCP	73	Phaco ECCE ICCE	–	0.5 (1 month to 1 year)	Perforation (2/15; 13.3%), progressive surface disease (6/15; 40%)
Ram et al. (12)	21	SS	Group 1: 61 ± 8 Group 2: 61 ± 10	ECCE and ICCE	CFCF-1.3	Group 1: 0.5 Group 2: 0.3	Group 1: post-operative endophthalmitis (3/21; 14.2%), peripheral keratolysis (4/21; 19%) Group 2: filamentary keratitis (6/21; 28.5%) peripheral keratolysis (2/21; 9.5%)

*BCVA, best corrected visual acuity; SJS, Stevens-Johnson syndrome; CED, corneal epithelial defect; Phaco, phacoemulsification; ECCE, extracapsular cataract extraction; PCR, posterior capsular rupture; GVHD, graft-versus-host disease; CME, cystoid macular edema; OCP, ocular cicatricial pemphigoid; SPK, superficial punctate keratitis; SS, Sjogren's syndrome; ICCE, intracapsular cataract extraction*.

The main strength of the study lies in its sample size and the standardized and digitized way of data entry. The main limitation of the study is its retrospective design and the lack of data on scores based on questionnaires and objective tests like tear osmolarity and non-invasive tear breakup time (NITBUT), which could help correlate the severity of DED preoperatively to visual outcomes following cataract surgery. Also, inclusion of a control group comprising eyes that underwent routine cataract surgery without pre-existing DED or other ocular surface co-morbidities would have added value to this study. However, the lack of such a control group is another limitation of this study. It would be interesting to plan a prospective study in the future including the subjective and objective assessment of DED both before and after cataract surgery to understand the impact of cataract surgery on pre-existing DED and vice versa.

In conclusion, this study found that the visual outcomes after cataract surgery in DED were satisfactory, across different etiologies. Pre-existing keratopathy, specifically corneal scarring, was the main determinant of sub-optimal visual outcomes. The study also found that there were no alarmingly high rates of intra- or post-operative complications and no sight-threatening issues with cataract surgery in DED. The findings of this study will not only help cataract surgeons in better prognostication for their patients but will also help patients in avoiding delaying cataract surgery because of the unreasonable fear of complications.

## Data Availability Statement

The raw data supporting the conclusions of this article will be made available by the authors, without undue reservation.

## Ethics Statement

The studies involving human participants were reviewed and approved by Institute Review Board, L V Prasad Eye Institute. Written informed consent to participate in this study was provided by the participant's legal guardian/next of kin. Written informed consent was obtained from the individual(s) for the publication of any potentially identifiable images or data included in this article.

## Author Contributions

PD, AD, and SB: concept and design of study or acquisition of data or analysis and interpretation of data. PD, AD, SS, and SB: drafting the article or revising it critically for important intellectual content and final approval of the version to be published. All authors contributed to the article and approved the submitted version.

## Conflict of Interest

The authors declare that the research was conducted in the absence of any commercial or financial relationships that could be construed as a potential conflict of interest.

## Abbreviations

DED: dry eye disease
BAK: benzalkonium chloride
EMR: electronic medical record system
FBUT: fluorescein breakup time
MGD: meibomian gland disease
SS: Sjogren's syndrome
CC: chronic cicatrizing conjunctivitis
BCVA: best corrected visual acuity
WHO: World Health Organization
K: keratometry readings
HPMC: hydroxy-propyl methylcellulose
PMMA: Polymethyl methacrylate
BCL: bandage contact lens
IQR: inter-quartile range
SJS: Stevens-Johnson syndrome
OCP: ocular cicatricial pemphigoid
GVHD: graft-versus-host disease
MSICS: manual small incision cataract surgery
ECCE: extracapsular cataract extraction
ICCE: intracapsular cataract extraction
PCR: posterior capsular rupture
DMD: Descemet's membrane detachment
ZD: zonular dialysis
IOL: intraocular lens
NSAID: non-steroidal anti-inflammatory drugs
NITBUT: non-invasive tear breakup time.
